# Acute febrile torticollis in youth: clinical investigation and current management

**DOI:** 10.11604/pamj.2015.21.163.5843

**Published:** 2015-06-25

**Authors:** Naouar Ouattassi, Mohammed Chmiel, Zakaria El Kerouiti, Mohammed Ridal, Mohammed Nouredine Alami

**Affiliations:** 1 ENT Head and Neck Surgery Department, Hassan II University Hospital, Fez, Morocco

**Keywords:** Fever, torticollis, children, parapharyngeal abscess

## Abstract

Acute febrile torticollis in children is a rare and a special clinical picture of variable causes. It may indicate an inflammatory or an infectious pathology affecting any of the anatomical structures of the neck. Treatment is quite clearly defined, and it may be a therapeutic emergency. It is a condition that all ENT specialists must be familiar with since they are most likely to be the first physician to whom such a child is brought

## Introduction

Torticollis is a vicious and permanent attitude of the head and the neck that could be painful or not. It is a rare symptom of various and less or more severe aetiologies. In a Pubmed literature review using the key-words “torticollis”, “fever” and “child” only five manuscripts have been found. A febrile torticollis is suspicious of infectious origin. In fact it might be related to potentially serious deep space neck infections which could compromise the airway or evolve toward a sepsis [1, 2]. However, inflammatory causes must not be omitted. The etiologic diagnosis is based on clinical suspicion and CT scan investigation. Bacteriology is essential for proper management. In our manuscript we discuss the clinical presentation particularities, the management strategy and the outcome of three children that have been presented to pediatric emergency department of Hassan II University Hospital with an acute febrile torticollis

## Patient and observation

### Case 1

**Figure 1:**
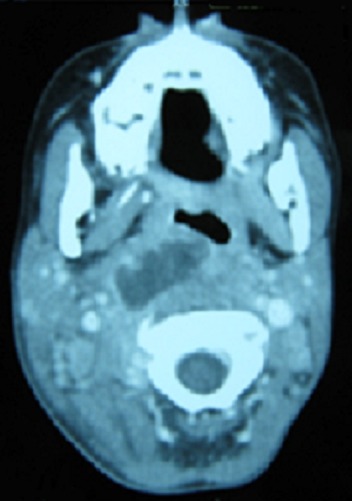
CT scan axial view that shows a right parapharyngeal abscess

An eight years old girl has presented to pediatric emergency with a recent history of febrile odynophagie subsequently complicated by a torticollis that has settled 5days after. Clinical examination has found a febrile child at 39°C and a bulging anterior pillar of the right tonsil without rash or conjunctival hyperemia. CT scan was performed and has revealed right peritonsillar and parapharyngeal abscess (Figure 1). At laboratory tests we found a white blood cell count (WBC) of 14000/mm^3^ and C - reactive protein (CRP) of 102mg / l. The child underwent a trans-oral drainage of the abscess under general anesthesia and airway protection. The bacteriological sampling objectified mixed unspecific flora sensitive to amoxicillin-clavulanic acid with no evidence of mycobacterium tuberculosis. Antibiotherapy was prescribed based on amoxicilline- clavulanic acid (80mg/kg a day) and aminoglycoside (3mg/kg a day for 5 days). The child was apyretic 48 hours after, and C- reactive protein 72 hours later was at 45 mg/l. Cervical spine traction was not necessary since the torticollis has been resolved after 5 days.

### Case 2

**Figure 2:**
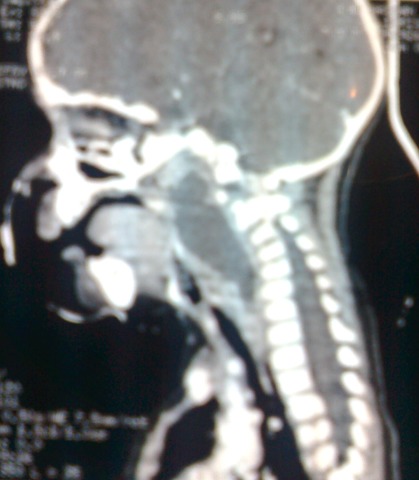
CT scan sagittal view that shows a retropharyngeal abscess with straightness of the cervical spine

A 2 years old male infant has presented to pediatric emergency with an acute febrile torticollis that has occurred 3 days after a rhinopharyngitis onset. Physical examination has found febrile and eupneic child who has a stable hemodynamic status. Physical examination has shown a bulging posterior wall of the oropharynx with several lenticular cervical lymph nodes. CT scan (Figure 2) has revealed a retropharyngeal abscess with straightness of the cervical spine. The child underwent a trans-oral drainage of the abscess under general anesthesia. According to bacteriological findings the patient has received intravenous antibiotherapy based on cephalosporin of 3rd generation (Ceftriaxone 50mg/kg a day) and aminoglycoside (3mg/Kg a day) with good outcome on both clinical and biological levels. The child was discharged from the hospital after a week. Antibiotherapy has been taken for three weeks. The straightness of the spine has been resolved after surgical drainage of the abscess.

### Case 3

**Figure 3:**
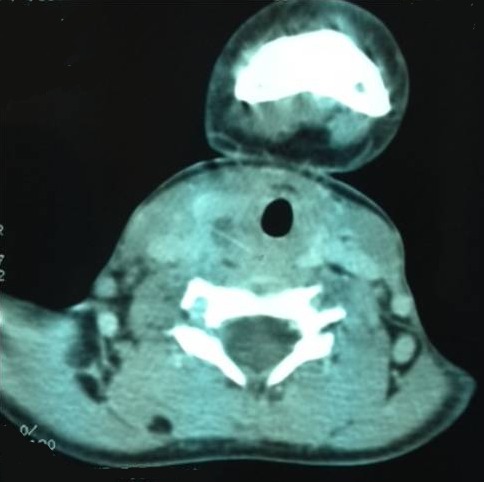
CT scan axial view that shows a right retro-thyroidien abscess and a foreign body (thorn: black arrow)

**Figure 4:**
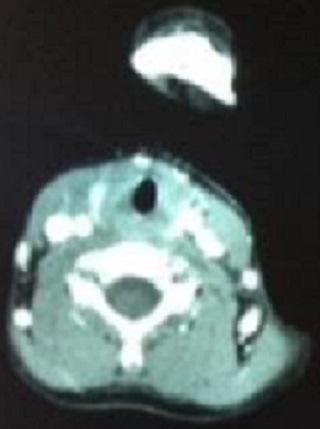
post operative CT scan

A 5 years old boy has presented to pediatric emergency with febrile odynophagia and torticollis that has occurred 2 days after he has eaten a prickly pear, no diarrhea or vomiting has been reported. The physical examination has found a febrile child with a right torticollis and no evidence of upper airways infection. Cervical and parapharyngeal CT scan (Figure 3) has revealed a foreign body that was a thorn with cervical abscess. At laboratory tests we found a WBC of 20000/mm3 and a CRP of 143mg/l. The child underwent surgical drainage of the abscess and removal of the thorn. Post operative course was uneventful. No miro-organism has been found in bacteriological samples, so the child received a wide spectrum antibiotic therapy based on cephalosporin of 3rd generation (Ceftriaxone 50mg/Kg a day) aminoglycoside (3mg/ Kg a day for 5 days) and metronidazole (40mg/Kg a day). Clinical, biological and radiological outcome was comforting (Figure 4). The patient was discharged from the hospital four days after. The antibiotics have been taken for 3 weeks.

## Discussion

Acute febrile torticollis is often seen in children presenting with inflammatory conditions of the upper respiratory tract and the neck. It is the result of an irritating process of cervical muscles, nerves or vertebrae that cause a unilateral muscle spasm responsible for the head posture. Posturing of the head occurs with unilateral spasm of the sternocleidomastoid muscle such that the child will position his head with the occiput rotated to the affected side and the chin rotated to the contra lateral side. At a physiopathological level it is known that retropharyngeal space is divided into anterior and posterior areas by the alar part of prevertebral fascia, the severe inflammation of the retropharyngeal lymph nodes in the anterior retropharyngeal space could form abscess or cellulitis that may lead to severe complications such mediastinitis. However, it is exceptional that the inflammation within the anterior area will spread to the prevertebral area [1]. Nevertheless, early diagnosis and treatment are to prevent these complications. Many authors agree that acute febrile torticollis due to retropharyngeal abscess occurs mainly (from 75 to 90% of cases) in youth under 5 years old [2, 3]. This might be explained by the fact that retropharyngeal space in youth is fairly open and becomes involuted with age, shrinking back after the age of three [1]. Thus, after acute pharyngitis, the retropharyngeal lymph nodes become inflamed and swollen leading to an inflammatory torticollis by irritating cervical muscles and nerves. Although febrile torticollis in adults are due to retropharyngeal abscess that occurs as a complication of an endoscopic procedure or ingestion of a foreign body such as fish bones in patients of specific conditions as underlying diseases (diabeties, steroids taking, HIV?.) [4–6], acute febrile torticollis in children is mainly of non traumatic aetiology. However, we report a case of laterocervical abscess due to a migrating vegetal foreign body. Through a biographical research over Pubmed we believe this is the first case of cervical abscess due to a migrating foreign body in children. Over eighty aetiology of acute torticollis all ages combined inflammatory and infectious conditions of the neck and the upper airways are the main causes in children. Thus diagnosis is based on imaging techniques. Although there are few artifices to suspect these conditions on standard radiographies [7], their sensitivity and specificity are limited not exceeding 40% [8]. 72% of pediatricians agree that CT scan is first exam to perform to disclose such aetiologies [2, 9]. Therefore, CT scan can assert the diagnosis of retropharyngeal abscess and it differential diagnosis with the retropharyngeal cellulitis. The correlation between CT appearance and intraoperative finding is significant with a false positive rate (CT shows an abscess that is not found at surgical exploration) of 10% and a false negative rate (abscess found during the surgery not revealed by CT) of 13% [2]. MRI might be interesting when rotatory subluxation of the atlantoaxial joint is suspected [10].

On microbiology, anaerobic bacteria (Bacteroides, Peptostreptococcus and Fusobacterium) are the most frequently incriminated. However, their identification in bacteriological samples is quite difficult since they are fragile micro-organism that requires specific conditions of transportation and breeding ground. Therefore cultures are often sterile. Also, few observations have reported a mixed flora (anaerobic bacteria and Staphylococcus aureus, Haemophilus influenza or Streptococcus βhemolytic A) [5, 6], others have found a pure aerobic flora (Staphylococcus aureus and Streptococcus βhemolytic A) [2]. We believe that culture on special breeding ground for mycobacteria ought to be demanded especially for mycobacterium tuberculosis particularly in endemic regions of tuberculosis. Retropharyngeal abscesses are a potentially serious infection of the deep neck spaces because of its extensive potential and its complications such as compression of the upper airway, sepsis, mediastinitis, spondylitis and epidural abscess [6]. However, the major complication is atlantoaxial subluxation described as Grisel′s syndrome following an inflammatory arthritis or tendonitis which can be life-threatening by a sudden spinal cord section [6]. The main differential diagnosis of retropharyngeal abscess or cellulitis is Kawasaki disease which is a systemic vascularitis of undetermined origin [9]. A literature search found a little more than 5 cases of Kawasaki disease with an initial diagnosis of retropharyngeal abscess confirmed by CT scan [6, 9]. In these cases, the outcome was good under intravenous immunoglobulin's treatment. Surgical exploration is not recommended [9]. Curative management of septic retropharyngeal abscess is based on three components: antibiotics, surgical drainage of the abscess and cervical spine traction. Although, the first one is unanimous, the last two are controversial. In fact, a 2002 survey showed that 22% of practitioners believe that surgical drainage is always appreciated, cons 31% that willingly would introduce probabilistic antibiotherapy in the absence of compression features of the upper airway [8]. In 2003 Frances and Craig study has shown over 14 patients similar results between patients who received antibiotics alone and those whom underwent surgical drainage and post operative antibiotics [1]. Also a 2004 study Al-Sabah, Ben Salleen et al. concluded that first intension antibiotics based on intravenous clindamycin is still effective and that surgical treatment is reserved for cases not responding to medical treatment [10]. This contrasts with recent reports of surgical treatment in 85% to 100% of cases [7]. However, authors agree that surgical drainage is required each time the infection is spread to the prevertebral space with a high risk of mediastinitis, spondylitis or epidural abscess or compression of the upper airway. Also, antibiotic therapy alone is considered in cases of retropharyngeal cellulitis or retropharyngeal small abscess when there is no risk to compromise the upper airway. Although simple cases can be solved within a few days of rest, some authors recommend cervical spine traction when torticollis does not resolve after few days of intravenous antibiotic therapy [10].

## Conclusion

Children with acute torticollis need careful evaluation for either overt or occult otolaryngologic infections. Computed tomography and magnetic resonance imaging are helpful in determining the cause of the acute torticollis and in ruling out rotatory subluxation of the atlanto-axial joint. In most cases it is a nonspecific infection of anaerobic or mixed flora. Nevertheless a specific infection such as tuberculosis or inflammatory cause such as Kawasaki disease should not be omitted. Although antibiotics are unanimous, surgical drainage and cervical spine traction are discussed according to the case.
